# Supplementary material for: Role and mechanism of NCAPD3 in promoting malignant behaviors in gastric cancer
Source: Front Pharmacol. 2024 Apr 22;15:1341039. doi: 10.3389/fphar.2024.1341039 (PMC11070777; doi:10.3389/fphar.2024.1341039)
Supplement: Supplementary file 11 [file DataSheet2.ZIP › GSEA/immunologic/my_analysis.Gsea.1599462003817/GSE19888_CTRL_VS_A3R_INHIBITOR_TREATED_MAST_CELL_DN.html]

Details for gene set GSE19888\_CTRL\_VS\_A3R\_INHIBITOR\_TREATED\_MAST\_CELL\_DN[GSEA]

|  || Dataset | filtered\_dataset.sample\_info.cls#WT\_versus\_NCAPD3\_MUT |
| Phenotype | sample\_info.cls#WT\_versus\_NCAPD3\_MUT |
| Upregulated in class | NCAPD3\_MUT |
| GeneSet | GSE19888\_CTRL\_VS\_A3R\_INHIBITOR\_TREATED\_MAST\_CELL\_DN |
| Enrichment Score (ES) | -0.41413376 |
| Normalized Enrichment Score (NES) | -2.4383047 |
| Nominal p-value | 0.0 |
| FDR q-value | 0.015805531 |
| FWER p-Value | 0.18 |
Table: GSEA Results Summary

  

Fig 1: Enrichment plot: GSE19888\_CTRL\_VS\_A3R\_INHIBITOR\_TREATED\_MAST\_CELL\_DN      
 Profile of the Running ES Score & Positions of GeneSet Members on the Rank Ordered List

  

| SYMBOL | TITLE | RANK IN GENE LIST | RANK METRIC SCORE | RUNNING ES | CORE ENRICHMENT || 1 | 51111 | KMT5B | 2 | 1.217 | 0.0433 | No |
| 2 | 23174 | ZCCHC14 | 27 | 1.004 | 0.0627 | No |
| 3 | 50861 | STMN3 | 126 | 0.777 | 0.0197 | No |
| 4 | 653390 | RRN3P2 | 171 | 0.707 | 0.0136 | No |
| 5 | 55599 | RNPC3 | 227 | 0.652 | -0.0026 | No |
| 6 | 152485 | ZNF827 | 268 | 0.618 | -0.0090 | No |
| 7 | 147929 | ZNF565 | 311 | 0.587 | -0.0181 | No |
| 8 | 1387 | CREBBP | 387 | 0.544 | -0.0529 | No |
| 9 | 64324 | NSD1 | 534 | 0.455 | -0.1428 | No |
| 10 | 9852 | EPM2AIP1 | 634 | 0.406 | -0.2002 | No |
| 11 | 84159 | ARID5B | 699 | 0.379 | -0.2330 | No |
| 12 | 80218 | NAA50 | 782 | 0.332 | -0.2807 | No |
| 13 | 5898 | RALA | 813 | 0.309 | -0.2912 | No |
| 14 | 50640 | PNPLA8 | 881 | -0.296 | -0.3293 | No |
| 15 | 571 | BACH1 | 884 | -0.300 | -0.3197 | No |
| 16 | 220213 | OTUD1 | 889 | -0.304 | -0.3114 | No |
| 17 | 694 | BTG1 | 901 | -0.326 | -0.3074 | No |
| 18 | 5329 | PLAUR | 952 | -0.373 | -0.3302 | No |
| 19 | 51646 | YPEL5 | 1014 | -0.413 | -0.3596 | No |
| 20 | 53349 | ZFYVE1 | 1018 | -0.417 | -0.3464 | No |
| 21 | 11152 | WDR45 | 1032 | -0.430 | -0.3401 | No |
| 22 | 9236 | CCPG1 | 1128 | -0.500 | -0.3911 | No |
| 23 | 604 | BCL6 | 1136 | -0.504 | -0.3776 | No |
| 24 | 153222 | CREBRF | 1187 | -0.559 | -0.3936 | Yes |
| 25 | 9665 | KIAA0430 | 1192 | -0.566 | -0.3756 | Yes |
| 26 | 7128 | TNFAIP3 | 1198 | -0.573 | -0.3582 | Yes |
| 27 | 2043 | EPHA4 | 1228 | -0.602 | -0.3572 | Yes |
| 28 | 9975 | NR1D2 | 1230 | -0.605 | -0.3357 | Yes |
| 29 | 10106 | CTDSP2 | 1239 | -0.618 | -0.3188 | Yes |
| 30 | 7057 | THBS1 | 1242 | -0.620 | -0.2974 | Yes |
| 31 | 26137 | ZBTB20 | 1244 | -0.622 | -0.2753 | Yes |
| 32 | 80853 | KDM7A | 1311 | -0.714 | -0.2972 | Yes |
| 33 | 5997 | RGS2 | 1336 | -0.770 | -0.2864 | Yes |
| 34 | 56605 | ERO1B | 1340 | -0.778 | -0.2599 | Yes |
| 35 | 113791 | PIK3IP1 | 1345 | -0.787 | -0.2339 | Yes |
| 36 | 133746 | JMY | 1350 | -0.793 | -0.2076 | Yes |
| 37 | 219539 | YPEL4 | 1354 | -0.803 | -0.1803 | Yes |
| 38 | 54790 | TET2 | 1364 | -0.842 | -0.1559 | Yes |
| 39 | 26959 | HBP1 | 1395 | -1.073 | -0.1383 | Yes |
| 40 | 1475 | CSTA | 1400 | -1.162 | -0.0984 | Yes |
| 41 | 51274 | KLF3 | 1406 | -1.296 | -0.0544 | Yes |
| 42 | 255394 | TCP11L2 | 1408 | -1.538 | 0.0015 | Yes |
Table: GSEA details [plain text format]

  

Fig 2: GSE19888\_CTRL\_VS\_A3R\_INHIBITOR\_TREATED\_MAST\_CELL\_DN      
 Blue-Pink O' Gram in the Space of the Analyzed GeneSet

  

Fig 3: GSE19888\_CTRL\_VS\_A3R\_INHIBITOR\_TREATED\_MAST\_CELL\_DN: Random ES distribution      
 Gene set null distribution of ES for **GSE19888\_CTRL\_VS\_A3R\_INHIBITOR\_TREATED\_MAST\_CELL\_DN**
